# Predicting Real-World Functioning in Schizophrenia: The Relative Contributions of Neurocognition, Functional Capacity, and Negative Symptoms

**DOI:** 10.3389/fpsyt.2021.639536

**Published:** 2021-03-19

**Authors:** Zixu Yang, Soon Hong Lee, Nur Amirah Abdul Rashid, Yuen Mei See, Justin Dauwels, Bhing Leet Tan, Jimmy Lee

**Affiliations:** ^1^Research Division, Institute of Mental Health, Singapore, Singapore; ^2^Lee Kong Chian School of Medicine, Nanyang Technological University, Singapore, Singapore; ^3^School of Electrical and Electronic Engineering, Nanyang Technological University, Singapore, Singapore; ^4^Department of Occupational Therapy, Institute of Mental Health, Singapore, Singapore; ^5^Health and Social Sciences, Singapore Institute of Technology, Singapore, Singapore; ^6^Department of Psychosis, Institute of Mental Health, Singapore, Singapore

**Keywords:** schizophrenia, real-world functioning, negative symptoms, neurocognition, functional capacity

## Abstract

Neurocognition and functional capacity are commonly reported predictors of real-world functioning in schizophrenia. However, the additional impact of negative symptoms, specifically its subdomains, i.e., diminished expression (DE) and avolition-apathy (AA), on real-world functioning remains unclear. The current study assessed 58 individuals with schizophrenia. Neurocognition was assessed with the Brief Assessment of Cognition in Schizophrenia, functional capacity with the UCSD Performance-based Skills Assessment (UPSA-B), and negative symptoms with the Negative Symptom Assessment-16. Real-world functioning was assessed with the Multnomah Community Ability Scale (MCAS) with employment status as an additional objective outcome. Hierarchical regressions and sequential logistic regressions were used to examine the associations between the variables of interest. The results show that global negative symptoms contribute substantial additional variance in predicting MCAS and employment status above and beyond the variance accounted for by neurocognition and functional capacity. In addition, both AA and DE predict the MCAS after controlling for cognition and functional capacity. Only AA accounts for additional variance in employment status beyond that by UPSA-B. In summary, negative symptoms contribute substantial additional variance in predicting both real-world functioning and employment outcomes after accounting for neurocognition and functional capacity. Our findings emphasize both DE and AA as important treatment targets in functional recovery for people with schizophrenia.

## Introduction

Schizophrenia is a potentially debilitating condition that causes functional decline, impeding an individual's ability to live an independent and fulfilling life. Functional recovery has been an important treatment goal (1), and interest in exploring factors influencing functioning has grown (2, 3).

One of the most robust findings is the association between neurocognition and real-world functioning (4–7), which has been supported in both cross-sectional and longitudinal studies (8, 9). Functional capacity, an objective measurement of an individual's ability to perform tasks that are needed in everyday life, has also been found to be associated with real-world functioning (10–13) and at least partially mediate the relationship between neurocognition and real-world functioning (7, 14–19).

A meta-analysis found negative symptoms to be significantly associated with functioning and mediate the relationship between neurocognition and functional outcome (20). Negative symptoms, a core feature of schizophrenia, are also shown to negatively impact real-world functioning in areas such as work performance, everyday living, and social functioning (13, 21, 22). However, the role of negative symptoms in predicting real-world functioning beyond neurocognition and functional capacity remains unclear. Some studies find its unique contribution in predicting real-world functioning, which is independent of neurocognition (23) and functional capacity (15, 19, 24–26), and some studies find no additional influence on real-world functioning (14, 15).

Furthermore, the current conceptualization of negative symptoms comprises two distinct subdomains: diminished expression (DE), consisting of blunted affect and alogia, and avolition-apathy (AA), which includes amotivation, asociality, and anhedonia (27–31). These two subdomains may have different impacts on real-world functioning (32, 33), but their additional contributions to real-world functioning above and beyond neurocognition and functional capacity have not been well-studied.

Therefore, the aim of the present study was to examine the relative contributions of neurocognition, functional capacity, and negative symptoms on real-world functioning. Additionally, we examined employment to provide another perspective of real-world functioning. We hypothesized that negative symptoms, specifically its subdomains of DE and AA, might have unique contributions to real-world functioning and employment in schizophrenia.

## Materials and Methods

### Participants

Fifty-eight community-dwelling individuals aged between 20 and 51 and diagnosed with schizophrenia were recruited from the outpatient clinics at the Institute of Mental Health, Singapore. The diagnosis was ascertained using the Structured Clinical Interview for DSM-IV (34). The exclusion criteria were as follows: a history of stroke, traumatic brain injury, and neurological disorders such as epilepsy. Ethics approval for the study was granted by the National Healthcare Group's Domain Specific Review Board (Singapore). Written informed consent was obtained from all participants after the study procedures were fully explained.

### Assessments

#### Neurocognition

Neurocognition was assessed using the Brief Assessment of Cognition in Schizophrenia (BACS), a well-established neuropsychological battery used to evaluate cognitive functioning, including verbal memory and learning, working memory, psychomotor function, verbal fluency, and executive function in patients with schizophrenia (35). A composite *Z*-score was calculated for subsequent analysis (36).

#### Functional Capacity

The UCSD Performance-Based Skills Assessment-Brief (UPSA-B) (37) was used to assess participants' capacity to perform skills necessary in daily life with tangible props to simulate real-world tasks. The UPSA-B demonstrates adequate psychometric properties, which is close to the full version of the UPSA, and consists of two of the five subscales from the full UPSA (12): (a) financial skills and (b) communication skills.

#### Negative Symptoms

Negative symptoms were evaluated using the Negative Symptom Assessment-16 (NSA-16), which assesses the negative symptoms of schizophrenia comprising of five factors: communication, emotion/affect, social involvement, motivation, and retardation (38). The AA score was calculated by the sum of motivation and social involvement domain scores; DE score was obtained by the sum of communication and emotion/affect domain scores (39).

#### Real-World Functioning

Real-world functioning was assessed using the Multnomah Community Ability Scale (MCAS) (40, 41), a 17-item, clinician-rated tool that is used to evaluate broad dimensions of community functioning. The MCAS was the most frequently nominated scale of real-world outcome in the Validation of Everyday Real-World Outcomes (VALERO) in Schizophrenia project and is often used in the evaluation of community mental health interventions (42, 43).

Participant's employment status in the past 1 month was collected via self-report. It was initially recorded in three categories: unemployed, sheltered employment, and non-sheltered employment. Considering that the sheltered employment was non-competitive employment, this category was combined with the unemployed category. A binary variable (employed vs. unemployed) was created for analyses.

### Data Analysis

Prior to conducting any analysis, normality assumption was checked and satisfied for all continuous variables. Bivariate correlations between neurocognition, functional capacity, negative symptoms, AA, DE, and real-world functioning were performed using Pearson's correlation.

Demographic variables, including age, gender, and years of education, were individually examined in univariate regressions as a predictor of MCAS; none of them were significant and were excluded in the subsequent analysis as covariates. To test whether negative symptoms would explain additional variance in real-world functioning indexed by MCAS above and beyond the variance explained by neurocognition and functional capacity, hierarchical multiple regression was performed as follows: BACS was entered into the model in step one, followed by UPSA-B in step two. The NSA-16 total was entered in the final step. To examine which negative symptom subdomains influence real-world functioning, AA and DE were entered in the multiple regression model together with BACS and UPSA-B. Collinearity was checked for all multiple regression analysis.

For selecting predictors of employment status, demographic variables (i.e., age, gender, and years of education), neurocognition (BACS), functional capacity (UPSA-B), and negative symptom variables (NSA-16 total, AA and DE) were individually examined in univariate logistic regression. All demographic variables and neurocognition were not significant predictors of employment; hence, they were excluded in the subsequent analysis. Two sequential binary logistic regressions were conducted to assess whether negative symptoms or its subdomains would add unique variance in predicting employment status in addition to the variance explained by functional capacity. In both logistic regressions, functional capacity (UPSA-B) was entered in step 1 and then followed by either negative symptom (NSA-16 total score) or negative symptom subdomains (AA and DE) in step 2.

The *p*-values reported in this study are not adjusted for multiple comparisons because the analyses were planned and conducted in stages to test the hypothesis that negative symptoms and its subdomains may have unique contributions to real-world functioning and employment in schizophrenia. In addition, corrections might increase the likelihood of type II error (44–46). All analyses were performed with IBM SPSS Statistics for Windows, Version 25.0.

## Results

### Sample Characteristics

The sample characteristics are reported in Table 1. Three participants were not prescribed an antipsychotic at the time of assessment.

**Table 1:** Demographics and clinical data of study sample.

**Measure**	**Mean (SD)**	***N* (%)**
Age, years	31.48 (7.65)	
Total years of education	13.69 (2.72)	
Duration of illness, years	9.09 (7.22)	
Antipsychotic dose, mg^a^	431.74 (341.81)	
**Gender**		
Female		31 (53.40)
Male		27 (46.60)
**Marital status**		
Single/unmarried		54 (93.10)
Married		1 (1.72)
Separated/divorced		3 (5.17)
**Ethnicity**		
Chinese		50 (86.21)
Malay		5 (8.62)
Indian		3 (5.17)
**Employment status**		
Unemployed		31 (53.45)
Sheltered employment		4 (6.90)
Employed		23 (39.65)
BPRS-18 total	32.16 (8.98)	
MCAS	68.45 (7.75)	
BACS composite *Z*-score	−1.76 (1.28)	
UPSA-B	60.41 (15.15)	
NSA-16 total	40.79 (9.25)	
Communication	7.79 (3.32)	
Emotion/affect	8.53 (1.96)	
Social involvement	9.05 (2.61)	
Motivation	11.69 (2.68)	33
Retardation	3.72 (1.78)	
AA	20.74 (4.72)	
DE	16.33 (4.80)	

*BPRS-18, 18-item Brief Psychiatric Rating Scale; MCAS, Multnomah Community Ability Scale; BACS, Brief Assessment of Cognition in Schizophrenia; UPSA-B, UCSD Performance-based Skills Assessment—Brief; NSA-16, Negative Symptom Assessment-16; AA, Avolition-Apathy; DE, Diminished Expression*.

a *n = 55, total daily antipsychotic dose in chlorpromazine equivalents*.

### Correlations Among Cognition, Functional Capacity, Negative Symptoms, and Real-World Functioning

Bivariate intercorrelations among the measures are presented in Table 2. As expected, there was a moderate correlation between BACS and UPSA-B. MCAS was moderately correlated to both BACS and UPSA-B and inversely correlated to NSA-16.

**Table 2:** Correlations among variables.

	**MCAS**	**UPSA-B**	**BACS**	**NSA-16 Total**	**AA**	**DE**
MCAS	-					
UPSA-B	0.384 (0.003)	-				
BACS	0.355 (0.006)	0.459 (<0.001)	-			
NSA-16 Total	−0.684 (<0.001)	−0.393 (0.002)	−0.502 (<0.001)	-		
AA	−0.643 (<0.001)	−0.343 (0.008)	−0.403 (0.002)	0.820 (<0.001)	-	
DE	−0.547 (<0.001)	−0.397 (0.002)	−0.443 (0.001)	0.878 (<0.001)	0.481 (<0.001)	-

*Values in cells represent r (p). MCAS, Multnomah Community Ability Scale; UPSA-B, UCSD Performance-based Skills Assessment—Brief; BACS, Brief Assessment of Cognition in Schizophrenia; NSA-16, Negative Symptom Assessment-16; AA, Avolition-Apathy; DE, Diminished Expression*.

### Association With Real-World Functioning

As shown in Table 3, BACS alone significantly predicted MCAS, *F*_(1, 56)_ = 8.098, *p* = 0.006, accounting for 11% of the variance. After including UPSA-B in the model, the total variance accounted for significantly increased to 16% (*p* for *F* change = 0.045), and UPSA-B became the only significant predictor, *F*_(2, 55)_ = 6.382, *p* = 0.003. When the NSA-16 total score was added to the model, it was the only significant predictor and contributed an additional 30% variance in predicting MCAS. With all three predictors in the equation, the total variance accounted for reached 46%, *F*_(3, 54)_ = 16.916, *p* < 0.001.

**Table 3:** Hierarchical regression analysis for variables predicting real-world functioning indexed by MCAS.

**Step**	**Variable**	**β**	***t***	***p***	**Adjusted *R*^**2**^**	**Δ*R*^**2**^**	**Δ*F***	***p* (Δ*F*)**
1	BACS	0.355	2.846	0.006	0.111	0.126	8.098	0.006
2	BACS	0.227	1.660	0.103	0.159	0.062	4.204	0.045
	UPSA-B	0.280	2.050	0.045				
3	BACS	−0.035	−0.295	0.769	0.456	0.296	31.017	<0.001
	UPSA-B	0.148	1.313	0.195				
	NSA-16 Total	−0.644	−5.569	<0.001				

*MCAS, Multnomah Community Ability Scale; BACS, Brief Assessment of Cognition in Schizophrenia; UPSA-B, UCSD Performance-based Skills Assessment—Brief; NSA-16, Negative Symptom Assessment-16*.

When AA and DE were entered into the multiple regression model together with BACS and UPSA-B scores to predict MCAS, a total of 46% of variance was explained, *F*_(4, 53)_ = 13.140, *p* < 0.001; *R*^2^ = 0.498. Both negative subdomains (i.e., AA and DE) were significant predictors, while BACS and UPSA-B were not (Table 4).

**Table 4:** Multiple regression analysis for variables predicting real-world functioning indexed by MCAS.

	**Std. Error**	**β**	***t***	***p***	***sr^**2**^***
BACS	0.713	−0.012	−0.104	0.918	0.000
UPSA-B	0.058	0.117	1.025	0.310	0.010
AA	0.189	−0.474	−4.117	<0.001	0.161
DE	0.192	−0.279	−2.344	0.023	0.052

*MCAS, Multnomah Community Ability Scale; BACS, Brief Assessment of Cognition in Schizophrenia; UPSA-B, UCSD Performance-based Skills Assessment—Brief; AA, Avolition-Apathy; DE, Diminished Expression; sr2, semi-partial correlation*.

### Association With Employment

In the first sequential logistic regression, the likelihood ratio test of block 1 was significant when UPSA-B was the only predictor, χ^2^ (df = 1) = 5.030, *p* = 0.025. After adding the NSA-16 total score in step 2, the likelihood ratio test was significant, χ^2^ (df = 2) = 16.611, *p* < 0.001, and the deviance in employment accounted for was substantial, Nagelkerke *R*^2^ = 0.337. Comparison of likelihood ratios for models with and without the NSA-16 total score showed significant improvement with the addition of the NSA-16 total, χ^2^ (df = 1) = 11.582, *p* = 0.001. UPSA-B became a non-significant predictor in step 2, while a significant predictive effect of the negative symptom total score on employment status was observed.

In the second sequential logistic regression, AA and DE were entered into the model in step 2 following UPSA-B in step 1. The likelihood ratio test was significant for the final model, χ^2^ (df = 3) = 16.399, *p* = 0.001, with significant improvement made by AA and DE, χ^2^ (df = 2) = 11.370, *p* = 0.003. AA was the only significant predictor of employment status (Table 5).

**Table 5:** Logistic regression analyses for variables predicting employment status.

**Logistic regression**	**Step**	**Variable**	***B* (S.E.)**	**Wald**	**Odds ratio**	***p***
1	1	UPSA-B	0.042 (0.020)	4.536	1.043	0.033
	2	UPSA-B	0.028 (0.023)	1.472	1.028	0.225
		NSA-16 Total	−0.144 (0.049)	8.552	0.866	0.003
2	1	UPSA-B	0.042 (0.020)	4.536	1.043	0.033
	2	UPSA-B	0.025 (0.023)	1.138	1.025	0.286
		AA	−0.181 (0.084)	4.616	0.835	0.032
		DE	−0.136 (0.087)	2.437	0.873	0.119

*UPSA-B, UCSD Performance-based Skills Assessment—Brief; NSA-16, Negative Symptom Assessment-16; AA, Avolition-Apathy; DE, Diminished Expression*.

## Discussion

In this study, we investigated the additional influence of negative symptoms on real-world functioning indexed by both clinician-rated functioning scale and employment status after controlling for the effect of neuro cognition and functional capacity. We found that negative symptoms contributed a unique and substantial amount of variance to real-world functioning in people with schizophrenia.

In the present study, negative symptoms appear to be a key predictor of real-world functioning. Neurocognition and functional capacity became non-significant predictors of real-world functioning after negative symptoms were added in the model. This result is in contrast to previous studies, which attributed a large proportion of variance of real-world functioning to neurocognition and functional capacity (4, 11). On the other hand, there are studies supporting our findings. Vesterager et al. (47) found that only negative symptoms significantly predicted overall functioning and UPSA-B did not account for additional variance in real-world outcomes beyond that accounted for by negative symptoms. Negative symptoms, not UPSA, were consistently identified as useful predictors in areas of useful activities, self-care, and relationships (26).

Additionally, both AA and DE were significantly associated with real-world functioning after controlling for cognition and functional capacity. This is in contrast to previous reports that DE has no additional predictive value after controlling for AA (32, 48, 49). One recent study found amotivation to mediate the association between neurocognition and social functioning in individuals with psychotic disorder although DE had no such mediating effect (50). Meanwhile, we found that AA had a larger impact compared with DE (AA: SR^2^ = 0.161; DE SR^2^ = 0.052). This is congruent with previous reports that AA, especially avolition, has a better predictive ability for functioning than DE (48, 51–54).

The result for employment status was similar to that for MCAS. Functional capacity was significantly associated with employment status; however, when negative symptoms were added, the model improved substantially, and functional capacity was no longer a significant predictor. This result is in part supported by a previous finding that patients who were employed scored significantly higher on the UPSA-B than those who were not employed, but the effect was attenuated after including symptoms of psychosis (55).

In our study, AA was the key negative symptom domain associated with employment. The effects of AA and DE on employment is mixed in previous studies. Liemburg et al. (50) reported amotivation mediated the association between cognition and employment status in patients with recent onset psychotic illness although DE mediated the relationship in a longer illness duration cohort. Strauss et al. (51) reported that patients with AA symptoms were less likely to be gainfully employed and less likely to complete high-quality work than patients with DE symptoms. In line with our result, Ang et al. (56) showed that AA, not DE, was significantly associated with employment status, but the impact of neurocognition or functional capacity was not considered in their study.

Heterogeneity of negative symptoms and functional outcome, various measurement tools adopted, and different sample characteristics may account for the divergent findings regarding the relationship between negative symptoms, cognition, functional capacity, and functional outcome. A majority of past studies used a general psychiatric symptoms scale, such as the Positive and Negative Syndrome Scale (PANSS) (57), to assess negative symptoms, which may not capture all aspects of the construct of negative symptoms. Therefore, the impact of negative symptoms might have been underestimated.

One main strength of this study is that the functional outcomes were indexed by both interviewer-rated functioning level and employment status, an important and pragmatic functional milestone. In some previous studies, employment was indexed by scale ratings on functioning and not objectively collected employment status (16). Functional milestones may not be captured by global scores on everyday functioning scales (58). In addition, the accuracy of the ratings of patients' functioning level might be compromised by patients' poor insight (59), impaired cognitive function, and the presence of psychiatric symptoms (60). In contrast, functional milestones, such as employment status, allow for greater objectivity in assessing an important aspect of real-world functioning.

Some limitations of the study should be mentioned. First, this study had a relatively small sample size with slightly more females than males, which may impact the generalization of the results. Nevertheless, our findings appear consistent with related reports and lend weight to the substantive influence of negative symptoms on real-world functioning. In addition, our study found no effect of gender on real-world functioning and employment status. Second, NSA-16 covers four out of five subdomains of negative symptoms, i.e., blunted affect, alogia, asociality, and avolition, with anhedonia not addressed (39). Although missing the portion on anhedonia, AA still emerged as a strong predictor of real-world functioning in our study. The strong association seen between real-world functioning and AA could be partly due to overlapping behaviors assessed between the measurement scales, a common challenge faced by studies assessing negative symptoms based on behavioral information. However, our study had employment as an alternate outcome, which showed AA's consistent and significant influence across both measures of real-world functioning. Third, the specific impact of negative symptom subdomains on real-world functioning may vary depending on duration of the psychotic illness and the types of functioning, such as independent living, social functioning and occupational functioning (50). The current study evaluated only the overall functioning and employment status in a group of patients with an average 9-year duration of illness. More studies are needed to examine the specific effect of subdomain of negative symptoms on different functioning outcomes with the illness duration considered. Last, this study measured real-world functioning and employment attainment cross-sectionally. Future studies could examine how changes in AA or DE influence real-functioning changes or job tenure (61).

The current study shows a robust influence of both AA and DE on real-world functioning. The results highlight the importance of negative symptoms as unmet treatment needs, especially in the area of functional recovery. Psychiatric rehabilitation programs would do well to go beyond cognitive remediation and skills retraining to address negative symptoms, such as avolition and apathy to maximize an individual's chance at community integration and employment. Future research efforts should focus on addressing and ameliorating negative symptoms in individuals with schizophrenia.

## Data Availability Statement

The datasets generated in this article are not readily available because some participants do not allow the data from this study to be used in other research or transferred to other researchers. Requests to access the datasets should be directed to Jimmy Lee, jimmy_lee@imh.com.sg.

## Ethics Statement

The studies involving human participants were reviewed and approved by National Healthcare Group's Domain Specific Review Board, Singapore. Written informed consent to participate in this study was provided by the participants' legal guardian/next of kin.

## Author Contributions

ZY, JL, BT, and JD designed the study and were responsible for the project management. JL, BT, and JD obtained funding. ZY, SL, and JL performed the statistical analysis and drafted the manuscript. ZY, NA, and YS recruited participants and collected the data. All authors gave feedback on the first draft of the manuscript and approved the submitted manuscript.

## Conflict of Interest

The authors declare that the research was conducted in the absence of any commercial or financial relationships that could be construed as a potential conflict of interest.

## References

[1] Kern RS Glynn SM Horan WP Marder SR. Psychosocial treatments to promote functional recovery in schizophrenia. Schizophr Bull. (2009) 35:347–61. 10.1093/schbul/sbn177 19176470 PMC2659313

[2] Lahera G Galvez JL Sanchez P Martinez-Roig M Perez-Fuster JV Garcia-Portilla P. Functional recovery in patients with schizophrenia: recommendations from a panel of experts. BMC Psychiatry. (2018) 18:176. 10.1186/s12888-018-1755-2 29871616 PMC5989342

[3] Bechi M Bosia M Spangaro M Buonocore M Cavedoni S Agostoni G. Exploring functioning in schizophrenia: predictors of functional capacity and real-world behaviour. Psychiatry Res. (2017) 251:118–24. 10.1016/j.psychres.2017.02.019 28199909

[4] Bowie CR Harvey PD. Cognitive deficits and functional outcome in schizophrenia. Neuropsychiatr Dis Treat. (2006) 2:531–6. 10.2147/nedt.2006.2.4.531 19412501 PMC2671937

[5] Fu S Czajkowski N Rund BR Torgalsboen AK. The relationship between level of cognitive impairments and functional outcome trajectories in first-episode schizophrenia. Schizophr Res. (2017) 190:144–9. 10.1016/j.schres.2017.03.002 28302394

[6] Helldin L Mohn C Olsson A-K Hjärthag F. Neurocognitive variability in schizophrenia spectrum disorders: relationship to real-world functioning. Schizophr Res Cognit. (2020) 20:100172. 10.1016/j.scog.2020.100172 32090024 PMC7026276

[7] Nuechterlein KH Subotnik KL Green MF Ventura J Asarnow RF Gitlin MJ. Neurocognitive predictors of work outcome in recent-onset schizophrenia. Schizophr Bull. (2011) 37:S33–40. 10.1093/schbul/sbr084 21860045 PMC3160123

[8] Green MF Kern RS Braff DL Mintz J. Neurocognitive deficits and functional outcome in schizophrenia: are we measuring the “right stuff”? Schizophr Bull. (2000) 26:119–36. 10.1093/oxfordjournals.schbul.a033430 10755673

[9] Green MF Kern RS Heaton RK. Longitudinal studies of cognition and functional outcome in schizophrenia: implications for MATRICS. Schizophr Res. (2004) 72:41–51. 10.1016/j.schres.2004.09.009 15531406

[10] Szabo S Merikle E Lozano-Ortega G Powell L Macek T Cline S. Assessing the relationship between performance on the University of California performance skills assessment (UPSA) and outcomes in schizophrenia: a systematic review and evidence synthesis. Schizophr Res Treat. (2018) 2018:9075174. 10.1155/2018/9075174 30687553 PMC6327277

[11] Twamley EW Doshi RR Nayak GV Palmer BW Golshan S Heaton RK. Generalized cognitive impairments, ability to perform everyday tasks, and level of independence in community living situations of older patients with psychosis. Am J Psychiatry. (2002) 159:2013–20. 10.1176/appi.ajp.159.12.2013 12450950

[12] Patterson TL Goldman S McKibbin CL Hughs T Jeste DV. UCSD Performance-Based Skills Assessment: development of a new measure of everyday functioning for severely mentally ill adults. Schizophr Bull. (2001) 27:235–45. 10.1093/oxfordjournals.schbul.a006870 11354591

[13] Reichenberg A Feo C Prestia D Bowie CR Patterson TL Harvey PD. The course and correlates of everyday functioning in schizophrenia. Schizophr Res Cognit. (2014) 1:e47–52. 10.1016/j.scog.2014.03.001 25045625 PMC4097820

[14] Bowie CR Leung WW Reichenberg A McClure MM Patterson TL Heaton RK. Predicting schizophrenia patients' real-world behavior with specific neuropsychological and functional capacity measures. Biol Psychiatry. (2008) 63:505–11. 10.1016/j.biopsych.2007.05.022 17662256 PMC2335305

[15] Bowie CR Reichenberg A Patterson TL Heaton RK Harvey PD. Determinants of real-world functional performance in schizophrenia subjects: correlations with cognition, functional capacity, and symptoms. Am J Psychiatry. (2006) 163:418–25. 10.1176/appi.ajp.163.3.418 16513862

[16] Fujino H Sumiyoshi C Sumiyoshi T Yasuda Y Yamamori H Ohi K. Predicting employment status and subjective quality of life in patients with schizophrenia. Schizophr Res Cognit. (2015) 3:20–5. 10.1016/j.scog.2015.10.005 28740804 PMC5506698

[17] Ho JS Moore RC Davine T Cardenas V Bowie CR Patterson TL. Direct and mediated effects of cognitive function with multidimensional outcome measures in schizophrenia: the role of functional capacity. J Clin Exp Neuropsychol. (2013) 35:882–95. 10.1080/13803395.2013.828021 23984631 PMC3789849

[18] Strassnig MT Raykov T O'Gorman C Bowie CR Sabbag S Durand D. Determinants of different aspects of everyday outcome in schizophrenia: the roles of negative symptoms, cognition, and functional capacity. Schizophr Res. (2015) 165:76–82. 10.1016/j.schres.2015.03.033 25868935 PMC4437911

[19] Bowie CR Depp C McGrath JA Wolyniec P Mausbach BT Thornquist MH. Prediction of real-world functional disability in chronic mental disorders: a comparison of schizophrenia and bipolar disorder. Am J Psychiatry. (2010) 167:1116–24. 10.1176/appi.ajp.2010.09101406 20478878 PMC3694770

[20] Ventura J Hellemann GS Thames AD Koellner V Nuechterlein KH. Symptoms as mediators of the relationship between neurocognition and functional outcome in schizophrenia: a meta-analysis. Schizophr Res. (2009) 113:189–99. 10.1016/j.schres.2009.03.035 19628375 PMC2825750

[21] Fulford D Niendam TA Floyd EG Carter CS Mathalon DH Vinogradov S. Symptom dimensions and functional impairment in early psychosis: more to the story than just negative symptoms. Schizophr Res. (2013) 147:125–31. 10.1016/j.schres.2013.03.024 23587696 PMC3663589

[22] Erickson M Jaafari N Lysaker P. Insight and negative symptoms as predictors of functioning in a work setting in patients with schizophrenia. Psychiatry Res. (2011) 189:161–5. 10.1016/j.psychres.2011.06.019 21813183

[23] Gonzalez-Blanco L Garcia-Portilla MP Dal Santo F Garcia-Alvarez L de la Fuente-Tomas L Menendez-Miranda I. Predicting real-world functioning in outpatients with schizophrenia: role of inflammation and psychopathology. Psychiatry Res. (2019) 280:112509. 10.1016/j.psychres.2019.112509 31446217

[24] Leifker FR Bowie CR Harvey PD. Determinants of everyday outcomes in schizophrenia: the influences of cognitive impairment, functional capacity, and symptoms. Schizophr Res. (2009) 115:82–7. 10.1016/j.schres.2009.09.004 19775869

[25] Best MW Gupta M Bowie CR Harvey PD. A longitudinal examination of the moderating effects of symptoms on the relationship between functional competence and real world functional performance in Schizophrenia. Schizophr Res Cognit. (2014) 1:90–5. 10.1016/j.scog.2014.03.002 25267939 PMC4175398

[26] Menendez-Miranda I Garcia-Portilla MP Garcia-Alvarez L Arrojo M Sanchez P Sarramea F. Predictive factors of functional capacity and real-world functioning in patients with schizophrenia. Eur Psychiatry. (2015) 30:622–7. 10.1016/j.eurpsy.2014.12.011 25681175

[27] Blanchard JJ Cohen AS. The structure of negative symptoms within schizophrenia: implications for assessment. Schizophr Bull. (2006) 32:238–45. 10.1093/schbul/sbj013 16254064 PMC2632211

[28] Liemburg E Castelein S Stewart R van der Gaag M Aleman A Knegtering H. Two subdomains of negative symptoms in psychotic disorders: established and confirmed in two large cohorts. J Psychiatr Res. (2013) 47:718–25. 10.1016/j.jpsychires.2013.01.024 23472837

[29] Strauss GP Hong LE Gold JM Buchanan RW McMahon RP Keller WR. Factor structure of the brief negative symptom scale. Schizophr Res. (2012) 142:96–8. 10.1016/j.schres.2012.09.007 23062750 PMC3502636

[30] Kirkpatrick B Strauss GP Nguyen L Fischer BA Daniel DG Cienfuegos A. The brief negative symptom scale: psychometric properties. Schizophr Bull. (2011) 37:300–5. 10.1093/schbul/sbq059 20558531 PMC3044634

[31] Marder SR Galderisi S. The current conceptualization of negative symptoms in schizophrenia. World Psychiatry. (2017) 16:14–24. 10.1002/wps.20385 28127915 PMC5269507

[32] Rocca P Montemagni C Zappia S Piterà R Sigaudo M Bogetto F. Negative symptoms and everyday functioning in schizophrenia: a cross-sectional study in a real world-setting. Psychiatry Res. (2014) 218:284–9. 10.1016/j.psychres.2014.04.018 24814140

[33] Gur RE Kohler CG Ragland JD Siegel SJ Lesko K Bilker WB. Flat affect in schizophrenia: relation to emotion processing and neurocognitive measures. Schizophr Bull. (2006) 32:279–87. 10.1093/schbul/sbj041 16452608 PMC2632232

[34] Association AP. Diagnostic Criteria from DSM-IV-TR. Washington, DC: American Psychiatric Pub (2000).

[35] Keefe RSE Poe M Walker TM Harvey PD. The relationship of the brief assessment of cognition in schizophrenia (BACS) to functional capacity and real-world functional outcome. J Clin Exp Neuropsychol. (2006) 28:260–9. 10.1080/13803390500360539 16484097

[36] Keefe RS Goldberg TE Harvey PD Gold JM Poe MP Coughenour L. The brief assessment of cognition in schizophrenia: reliability, sensitivity, and comparison with a standard neurocognitive battery. Schizophr Res. (2004) 68:283–97. 10.1037/t38021-000 15099610

[37] Mausbach BT Harvey PD Goldman SR Jeste DV Patterson TL. Development of a brief scale of everyday functioning in persons with serious mental illness. Schizophr Bull. (2007) 33:1364–72. 10.1093/schbul/sbm014 17341468 PMC2779885

[38] Axelrod BN Goldman RS Alphs LD. Validation of the 16-item negative symptom assessment. J Psychiatr Res. (1993) 27:253–8. 10.1016/0022-3956(93)90036-2 7905033

[39] Daniel DG. Issues in selection of instruments to measure negative symptoms. Schizophr Res. (2013) 150:343–5. 10.1016/j.schres.2013.07.005 23899996

[40] Barker S Barron N McFarland BH Bigelow DA. A community ability scale for chronically mentally ill consumers: part I. Reliability and validity. Community Ment Health J. (1994) 30:363–83. 10.1007/BF02207489 7956112

[41] Barker S Barron N McFarland BH Bigelow DA Carnahan T. A community ability scale for chronically mentally ill consumers: part II. Applications. Community Ment Health J. (1994) 30:459–72. 10.1007/BF02189063 7851100

[42] Tan BL Ng WY Sudhasan J Chng T Mok I Lee J. Factors associated with changes in community ability and recovery after psychiatric rehabilitation: a retrospective study. Community Ment Health J. (2018) 54:1221–7. 10.1007/s10597-018-0249-5 29480381

[43] Durbin J Dewa C Aubry T Rourke S. The use of multnomah community ability scale as a program evaluation tool. Can J Program Eval. (2004) 19:135–57.

[44] Rothman KJ. No adjustments are needed for multiple comparisons. Epidemiology (Cambridge, Mass). (1990) 1:43–6. 10.1097/00001648-199001000-00010 2081237

[45] Perneger TV. What's wrong with Bonferroni adjustments. BMJ. (1998) 316:1236. 10.1136/bmj.316.7139.1236 10.1136/bmj.316.7139.1236 9553006 PMC1112991

[46] Streiner DL Norman GR. Correction for multiple testing: is there a resolution? Chest. (2011) 140:16–8. 10.1378/chest.11-0523 21729890

[47] Vesterager L Christensen TØ Olsen BB Krarup G Melau M Forchhammer HB. Cognitive and clinical predictors of functional capacity in patients with first episode schizophrenia. Schizophr Res. (2012) 141:251–6. 10.1016/j.schres.2012.08.023 23017825

[48] Foussias G Mann S Zakzanis KK van Reekum R Agid O Remington G. Prediction of longitudinal functional outcomes in schizophrenia: the impact of baseline motivational deficits. Schizophr Res. (2011) 132:24–7. 10.1016/j.schres.2011.06.026 21771567

[49] Fervaha G Foussias G Agid O Remington G. Motivational and neurocognitive deficits are central to the prediction of longitudinal functional outcome in schizophrenia. Acta Psychiatr Scand. (2014) 130:290–9. 10.1111/acps.12289 24850369

[50] Liemburg EJ Enriquez-Geppert S Wardenaar KJ Bruggeman R Aleman A. Expressive deficits and amotivation as mediators of the associations between cognitive problems and functional outcomes: results from two independent cohorts. Schizophr Res. (2020) 218:283–91. 10.1016/j.schres.2019.12.018 31948899

[51] Strauss GP Horan WP Kirkpatrick B Fischer BA Keller WR Miski P. Deconstructing negative symptoms of schizophrenia: avolition-apathy and diminished expression clusters predict clinical presentation and functional outcome. J Psychiatr Res. (2013) 47:783–90. 10.1016/j.jpsychires.2013.01.015 23453820 PMC3686506

[52] Foussias G Remington G. Negative symptoms in schizophrenia: avolition and Occam's razor. Schizophr Bull. (2010) 36:359–69. 10.1093/schbul/sbn094 18644851 PMC2833114

[53] Galderisi S Bucci P Mucci A Kirkpatrick B Pini S Rossi A. Categorical and dimensional approaches to negative symptoms of schizophrenia: focus on long-term stability and functional outcome. Schizophr Res. (2013) 147:157–62. 10.1016/j.schres.2013.03.020 23608244

[54] Galderisi S Rossi A Rocca P Bertolino A Mucci A Bucci P. The influence of illness-related variables, personal resources and context-related factors on real-life functioning of people with schizophrenia. World Psychiatry. (2014) 13:275–87. 10.1002/wps.20167 25273301 PMC4219069

[55] Mausbach BT Harvey PD Pulver AE Depp CA Wolyniec PS Thornquist MH. Relationship of the brief UCSD Performance-based skills assessment (UPSA-B) to multiple indicators of functioning in people with schizophrenia and bipolar disorder. Bipolar Disord. (2010) 12:45–55. 10.1111/j.1399-5618.2009.00787.x 20148866 PMC2846793

[56] Ang MS Rekhi G Lee J. Vocational profile and correlates of employment in people with schizophrenia: the role of avolition. Front Psychiatry. (2020) 11:e00856. 10.3389/fpsyt.2020.00856 33192630 PMC7481460

[57] Kay SR Fiszbein A Opler LA. The positive and negative syndrome scale (PANSS) for schizophrenia. Schizophr Bull. (1987) 13:261–76. 10.1093/schbul/13.2.261 3616518

[58] Harvey PD Sabbag S Prestia D Durand D Twamley EW Patterson TL. Functional milestones and clinician ratings of everyday functioning in people with schizophrenia: overlap between milestones and specificity of ratings. J Psychiatr Res. (2012) 46:1546–52. 10.1016/j.jpsychires.2012.08.018 22979993 PMC3485423

[59] Gould F Sabbag S Durand D Patterson TL Harvey PD. Self-assessment of functional ability in schizophrenia: milestone achievement and its relationship to accuracy of self-evaluation. Psychiatry Res. (2013) 207:19–24. 10.1016/j.psychres.2013.02.035 23537844 PMC3640769

[60] Sabbag S Twamley EW Vella L Heaton RK Patterson TL Harvey PD. Predictors of the accuracy of self assessment of everyday functioning in people with schizophrenia. Schizophr Res. (2012) 137:190–5. 10.1016/j.schres.2012.02.002 22386735 PMC3351672

[61] Velligan DI Alphs L Lancaster S Morlock R Mintz J. Association between changes on the negative symptom assessment scale (NSA-16) and measures of functional outcome in schizophrenia. Psychiatry Res. (2009) 169:97–100. 10.1016/j.psychres.2008.10.009 19664826

